# Australasian hidradenitis suppurativa management guidelines

**DOI:** 10.1111/ajd.14388

**Published:** 2024-11-22

**Authors:** John Frew, Annika Smith, Pablo Fernandez Penas, Elisabeth Ellis, Peter Foley, Diana Rubel, Erin McMeniman, Gillian Marshman, Helen Saunders, Emma Veysey, Jenny Nicolopolous, Linda Spelman, Kurt Gebauer

**Affiliations:** ^1^ The Skin Hospital Sydney New South Wales Australia; ^2^ Department of Dermatology, Liverpool Hospital Sydney New South Wales Australia; ^3^ University of New South Wales Sydney New South Wales Australia; ^4^ Department of Dermatology, Westmead Hospital Sydney New South Wales Australia; ^5^ University of Sydney Sydney New South Wales Australia; ^6^ Flinders Medical Centre Adelaide South Australia Australia; ^7^ Skin Health Institute Melbourne Melbourne Victoria Australia; ^8^ St Vincent's Hospital Melbourne Victoria Australia; ^9^ Canberra Hospital Canberra Australian Capital Territory Australia; ^10^ Princess Alexandria Hospital Brisbane Queensland Australia; ^11^ Flinders University Adelaide South Australia Australia; ^12^ Royal Melbourne Hospital Melbourne Victoria Australia; ^13^ Queensland Institute of Dermatology Brisbane Queensland Australia; ^14^ Fremantle Dermatology Fremantle Western Australia Australia

**Keywords:** acne Inversa, consensus, guidelines, hidradenitis suppurativa

## Abstract

Hidradenitis Suppurativa is a burdensome inflammatory skin disease with significant quality of life impact. These management guidelines were developed to direct appropriate clinical management in the Australasian context. A systematic review was used for the basis of the consensus guidelines. Thirteen clinical experts were involved in a modified Delphi consensus process to develop the guidelines and treatment algorithms. Overall management strategies include appropriate severity assessment of disease and comorbidities, multimodal therapy with systemic and local treatments, and evidence‐based progression along the therapeutic ladder in the event of inadequate response. Sequential monotherapy with antibiotics and/or single agent therapy is discouraged and aggressive treatment of moderate to severe disease to capture the window of opportunity is highly emphasised. Specific considerations in the setting of disease comorbidities, pregnancy and breastfeeding are also addressed. Overall, the complex nature of HS requires a complex and multimodal therapeutic response with medical, physical and surgical therapies to achieve best patient outcomes.

## INTRODUCTION

Hidradenitis Suppurativa (HS) is a chronic inflammatory skin disease characterised by painful, purulent nodules, abscesses and tunnels with a predilection to the flexural regions of skin and a dramatic impact upon quality of life.^1^, ^2^ The pathogenesis is driven by dysregulated innate immunity, autoinflammation and alterations to epithelial stem cell fate in the pilosebaceous unit.^1^, ^3^, ^4^ Genetic factors account for up to 70% of the underlying cause of HS.^8^ These genetic factors include polymorphisms associated with epithelial stem cell fate (SOX9) and innate immune dysregulation. (KLF5, INAVA)^5^ and rarely monogenic associations with the gamma secretase complex and associated autoinflammatory syndromes (PTSPIP1, MFEV).^6^, ^7^ HS is strongly associated with hormonal dysregulation in conditions such as PCOS,^8^ insulin resistance^9^ and other inflammatory comorbidities such as inflammatory bowel disease^10^ and spondyloarthropathy.^11^ Existing management guidelines focus upon the use of systemic antibiotics and surgical monotherapy with biologic therapy considered for treatment of severe treatment resistant disease only.^12^, ^13^, ^14^, ^15^ Since 2018, a number of novel agents have been validated in Phase 3 randomised controlled trials providing a wide array of therapeutic options for patient management.^16^ It has also been acknowledged that early intervention with biologic therapy in moderate disease may help prevent progression to severe debilitating disease.^17^ Multimodal therapy combining medical and surgical therapy has emerged as providing superior disease control necessitating re‐orientation of recommendations.^18^, ^19^ Hence an updated Delphi consensus process^20^ was undertaken to develop up to date practical guidelines for management of HS in Australasia.

## METHODS

A total of 13 expert clinicians with clinical and research experience in HS were invited to take part in the consensus exercise. Preparatory activity included completion of a prospectively registered systematic review of the literature pertaining to the diagnosis, assessment, management and comorbidity screening of patients with HS (Search Strategy and PRISMA flow diagram presented as Figure S1). The results of this systematic review were presented to the expert clinicians as a series of statements for development of consensus via the modified Delphi method.^20^ Responses to these statements could include agree, disagree and free‐text comments suggesting changes were available. Consensus was defined as 70% or more of clinicians agreeing (or disagreeing) with the proposed statement.^20^ All suggestions in free text responses were analysed by thematic analysis and integrated into alterations based on qualitative methodology for future Delphi rounds. Rounds continued until consensus was achieved on all items. Statistical analysis was undertaken using Fleiss' kappa, and consensus stability was defined as a less than 15% variation in the degree of consensus between rounds.

## RESULTS

All 13 invited expert clinicians agreed to be involved in the consensus process. The preparatory systematic review was prospectively registered (INPLASY202430041) and conducted as outlined in Data S1. The 99 statements used for the consensus rounds are included as Data S1. All data from the literature was assessed for level of evidence using the GRADE criteria^21^ as previously described. The level of evidence for each identified therapy is presented in Data S1.

A total of three rounds were conducted to achieve consensus on all items. 100% expert clinicians responded to the first Delphi round, 11/13 (84.6%) responded to round 2 and 10/13 (77%) to round 3. Agreement increased from Round 1 to Round 3, with 94.8% of statements (92/99) achieving consensus in round 1, 96.9% (96/99) in round 2 and 100% (99/99) in Round 3. Consensus stability was achieved across the three rounds with increasing inter rater reliability from fair (Fleiss' kappa = 0.339) to moderate Fleiss' (kappa = 0.511) to substantial (Fleiss' kappa = 0.741) agreement with each progressive round.

Detailed data regarding free text responses is presented as Data S1.

The final consensus guidelines for the management of HS were approved by all participants and are presented as follows:

## DIAGNOSIS AND CLINICAL ASSESSMENT OF HIDRADENITIS SUPPURATIVA

Hidradenitis Suppurativa is diagnosed using the modified Dessau Criteria. Clinical Assessment is Made using the modified Hurley Sage or IHS4.

The diagnosis of HS is based upon the modified Dessau crtieria.^22^ This is defined as the presence of typical lesions (nodules, abscesses, tunnels, pseudo‐comedones) in typical areas (axillae, groin, sub‐mammary, buttocks), with 2 discrete areas involved within a 6‐month time period.^22^ (Table 1).

**TABLE 1:** Summary of Recommendations for diagnosis, clinical assessment and assessment outcomes in Hidradenitis Suppurativa.

Diagnosis	Description					Recommendation
Modified dessau criteria	Typical morphology (nodules, abscesses, sinus tracts, scars) Characteristic distribution or typography of lesions (intertriginous areas, axillae, inframammary folds, groins, buttocks, perianal and perineal areas) A relapsing, chronic disease course					Recommended
Severity grading	Description					Recommendation
Hurley staging	Stage I – abscess formation (single or multiple), no tunnels or cicatrization/scarring Stage II – recurrent abscesses with tunnels and scarring, single or multiple separated lesions Stage III – diffuse or almost diffuse involvement, or multiple interconnected sinus tracts and abscesses across the entire area					No Longer Recommended
AN Count	Abscesses + Nodules					No Longer Recommended
Refined hurley staging	1A: 1B: 1C 2A: 2B 2C 3	0–2 Body Areas >2 Body Areas >2 Body Areas <1% BSA <1% BSA <1% BSA >1% BSA	<5 Abscess/Nodules ≥5 Abscess/Nodules >5 Abscess/Nodules No Active Inflammation Inflammation <2 Body Areas; Inflammation >2 Body Areas	No Tunnels No Tunnels No Tunnels Tunnels Present Tunnels Present Tunnels Present Tunnels Present	Fixed Lesions Migratory Lesions	Recommended
IHS4	(Nodules x1) + (Abscesses x2) + (Tunnels x4)					Recommended
Assessment of clinical response	Description					Recommendation
HiSCR‐50	A 50% reduction in AN count with no increase in abscesses or draining tunnels					Not Recommended
HiSCR‐75	A 75% reduction in AN count with no increase in abscesses or draining tunnels					Recommended
HiSCR‐90	A 90% reduction in AN count with no increase in abscesses or draining tunnels					Recommended
IHS4‐55	A 55% reduction in the IHS4 score					Recommended
Change in AN Count	Absolute or % change in Abscess and Nodule count from baseline					Not Recommended
Change in dT Count	Absolute or % change in draining tunnel count from baseline					Not Recommended
Sartorius Score	Involved Region (3 points); nodule (2 points), tunnel (4 points), scar (1 point), longest distance between lesions. (0–8 points), presence of clear skin in each region (0–6 points)					Not Recommended
Change in Hurley Stage (Overall/ Regional)	*Not a dynamic score					Not Recommended
HASI‐R	Evaluation of severity of erythema, induration, tunnelling and surface area involved.					Not Recommended

There are currently no histological or serological diagnostic biomarkers to differentiate HS from other conditions. Many potential diagnostic markers have been proposed, however none have been appropriately validated for widespread clinical use.^23^ The diagnosis of HS should be made whilst considering other potential conditions which can have similar manifestations including cutaneous tuberculosis, donovanosis and folliculitis.^24^

The severity and activity of HS is assessed using a variety of different measures. Recommended severity assessments include the modified Hurley staging^25^ and the International Hidradenitis Suppurativa Severity Score System (IHS4).^26^ Previously utilised measures such as original Hurley staging^27^ and the Sartorius score^28^ are no longer recommended for routine clinical use. The modified Hurley stage provides a greater granularity as to the severity of disease and integrates the presence or absence of epithelialised tunnels which have both molecular and clinical relevance to therapeutic response.

Outcome measures for assessment of clinical response include the Hidradenitis Suppurativa Clinical Response (HiSCR^29^) 50, 75 and 90 and the IHS4‐55 outcome measures.^30^ The HiSCR75 and HiSCR90 are preferred given the relatively lower rates of placebo response which are inherently elevated in the HiSCR50.^31^, ^32^ Changes in hurley stage are not validated as response outcome variables and are not recommended. The suggested timeframe for assessment of clinical response is based on expert opinion. Whilst primary outcome measures in clinical trials are based at Week 12,^33^, ^34^, ^35^ guidelines recommend up to 24 weeks of therapy^36^ prior to treatment discontinuation due to lack of response.

Quality of life outcomes are very important given the enormous burden of disease in HS.^2^ Validated measures include the DLQI^2^ and the HiSQoL Questionnaire^37^

## OVERALL MANAGEMENT STRATEGY IN HIDRADENITIS SUPPURATIVA

Overarching Management Goals should include combined medical and surgical therapy with adequate management of inflammatory comorbidities and pain management.

**FIGURE 1:**
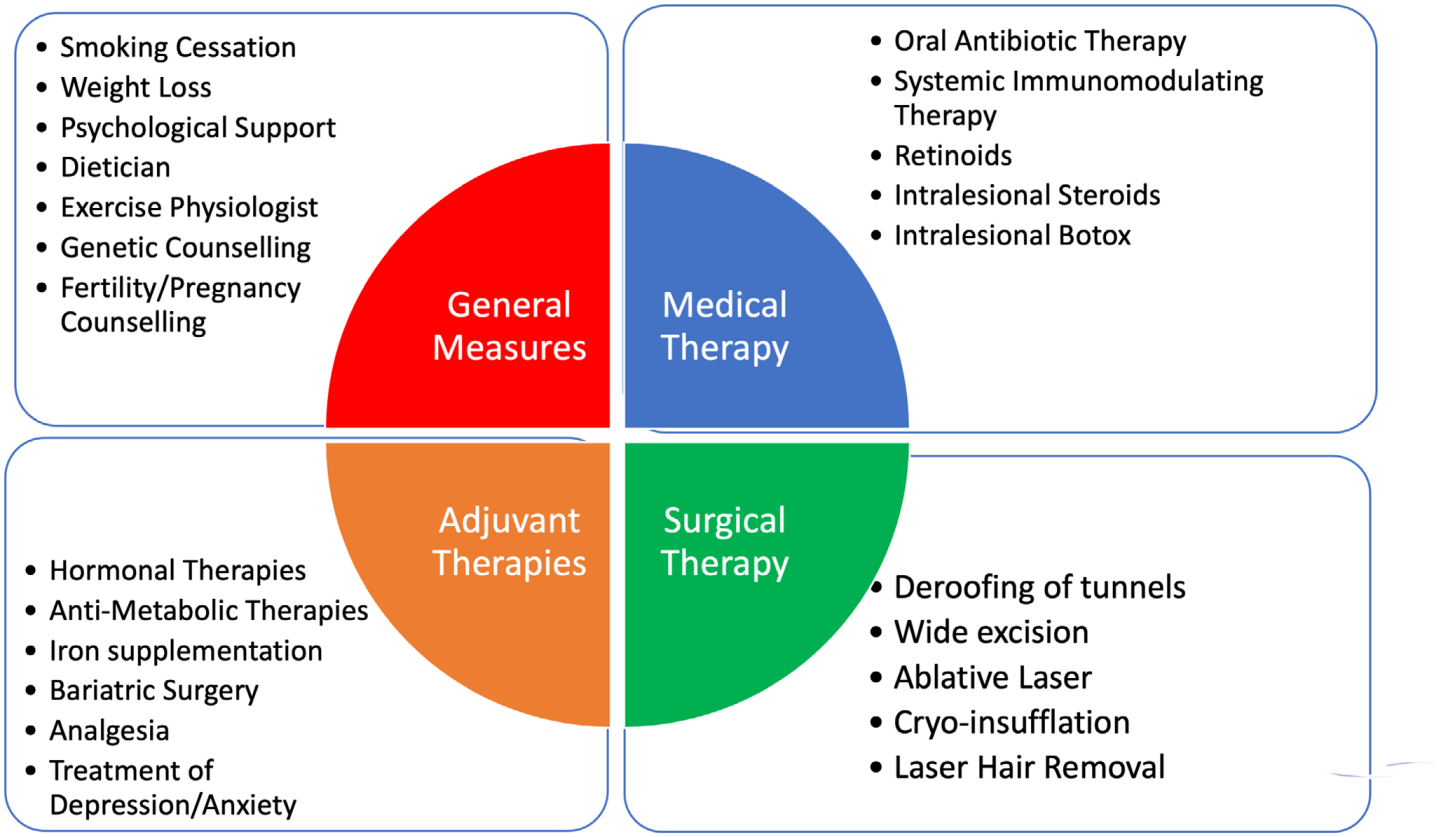
A schematic representation of the four major tenets of Hidradenitis Suppurativa management. Treatment under all four aspects are required to optimise disease control.

The four major tenets upholding the overall management strategy in HS include general measures, medical therapy, surgical therapy, and adjuvant therapies. (Figure 1). These general measures including smoking cessation^38^ and weight loss.^39^ These factors should be approached with a degree of sensitivity given the historical blame placed on patients in regard to the activity of their disease.^40^, ^41^ However, evidence suggests that weight loss and smoking cessation can increase the efficacy of systemic therapy as well as having overall health benefits.^38^ Given the strong genetic underpinnings of HS, genetic and fertility counselling may be appropriate,^42^ although no recommended genetic testing for HS is currently available. Psychological support may be required given the significant morbidity associated with the condition, as depression and anxiety are common.^43^ Medical therapy ranges from topical antiseptics,^44^ intermittent antibiotic therapy,^45^ hormonal inerventions,^46^ to systemic immunomodulatory therapy.^47^ As expanded upon below, other therapies such as retinoids^48^, ^49^ and traditional immunosuppressants^50^, ^51^ have fallen out of favour due to a lack of evidence and efficacy. Although in select patients they may provide some benefit to comorbid disease (for example, comedones or acne congolobata and retinoids^48^, ^49^). Adjuvant therapies to optimise baseline medical therapy include addressing coexistent comorbidities including polycystic ovarian syndrome,^52^ insulin resistance^53^ and obesity.^54^, ^55^ Surgical therapy combined with medical therapy has been shown to be safe and effective in multiple randomised controlled trials^58.59^ and provides benefit in targeting persistent tunnels and lesions less likely to respond to medical therapy alone.^56^ Pain control is an essential component to overall management.^57^, ^58^ An integrated multimodal therapeutic approach is recommended in HS to address the multiple causes of systemic inflammation.^59^, ^60^

## COMORBIDITY SCREENING IN HIDRADENITIS SUPPURATIVA

A number of comorbidities are strongly associated with HS. The utility of routine screening has not yet been appropriately validated. Appropriate management of comorbidities contributing to disease activity is mandatory, and screening in the presence of suggestive signs and symptoms should be directed by the clinician.

HS is associated with multiple inflammatory comorbidities.^61^ (Table 2) International guidelines recommend screening for a number of these comorbidities,^61^ although the utility of screening has not been formally evaluated.^62^ Some concern exists regarding the utility of screening for rare comorbidities (such as inflammatory eye conditions^63^) in HS, exemplified by the very high NNE (number needed to be exposed) as an indicator of the number of patients needing to be screened to identify one additional case of the disease.^62^

**TABLE 2:** Recommended Comorbidities for Screening in Hidradenitis Suppurativa.^61^, ^62^

Comorbidity in HS	Prevalence in HS	Number needed to screen	Level of evidence	Strength of recommendation
Acne vulgaris/conglobata	4.5%	N/A	II	B
Dissecting cellulitis of scalp	9.2%	N/A	II	B
Pilonidal cyst	32.6%	N/A	II	B
Pyoderma gangrenosum	0.18%	N/A	II	B
Depression	26.5%	108	II	B
Anxiety	18.1%	2169	II	B
Polycystic ovary syndrome	9%	N/A	II	B
Obesity	41.3%	N/A	II	B
Dyslipidemia	48.9%	12	II	B
Diabetes mellitus	10.6%	49	II	B
Metabolic syndrome	40%	N/A	II	B
Hypertension	24.4%	11	II	B
Cardiovascular disease	2.8%	18	II	B
Inflammatory bowel disease	1.8%	N/A	II	B
Spondyloarthritis	0.9%	N/A	II	B

Consensus recommendations include screening for the conditions presented in Table 2.

Conditions such as inflammatory bowel disease^10^ are best assessed through gastroscopy/colonoscopy given the presence of elevated faecal calprotectin and ASCA antibodies may be present in HS,^64^ particularly in severe disease. Inflammatory arthritis has been associated with HS particularly in the presence of HLAB27 positive serology.^65^, ^66^ The strong association of HS with cardiovascular and metabolic comorbidities independent of BMI and smoking status^61^ is reflected in the recommendation to screen for metabolic syndrome, although the clinical yield of screening for a variety of other psychological conditions and substance abuse was not universally recommended amongst experts.

## MANGAEMENT OF HIDRADENTIIS SUPPURATIVA: MILD–MODERATE DISEASE

Mild to Moderate Disease is defined as Hurley Stage 1,2a/2b.

Therapeutic options include topical/oral antibiotics, OCP/Spironolactone, with laser hair removal, local surgical intervention and intralesional botox/corticosteroids as additional options.

**FIGURE 2:**
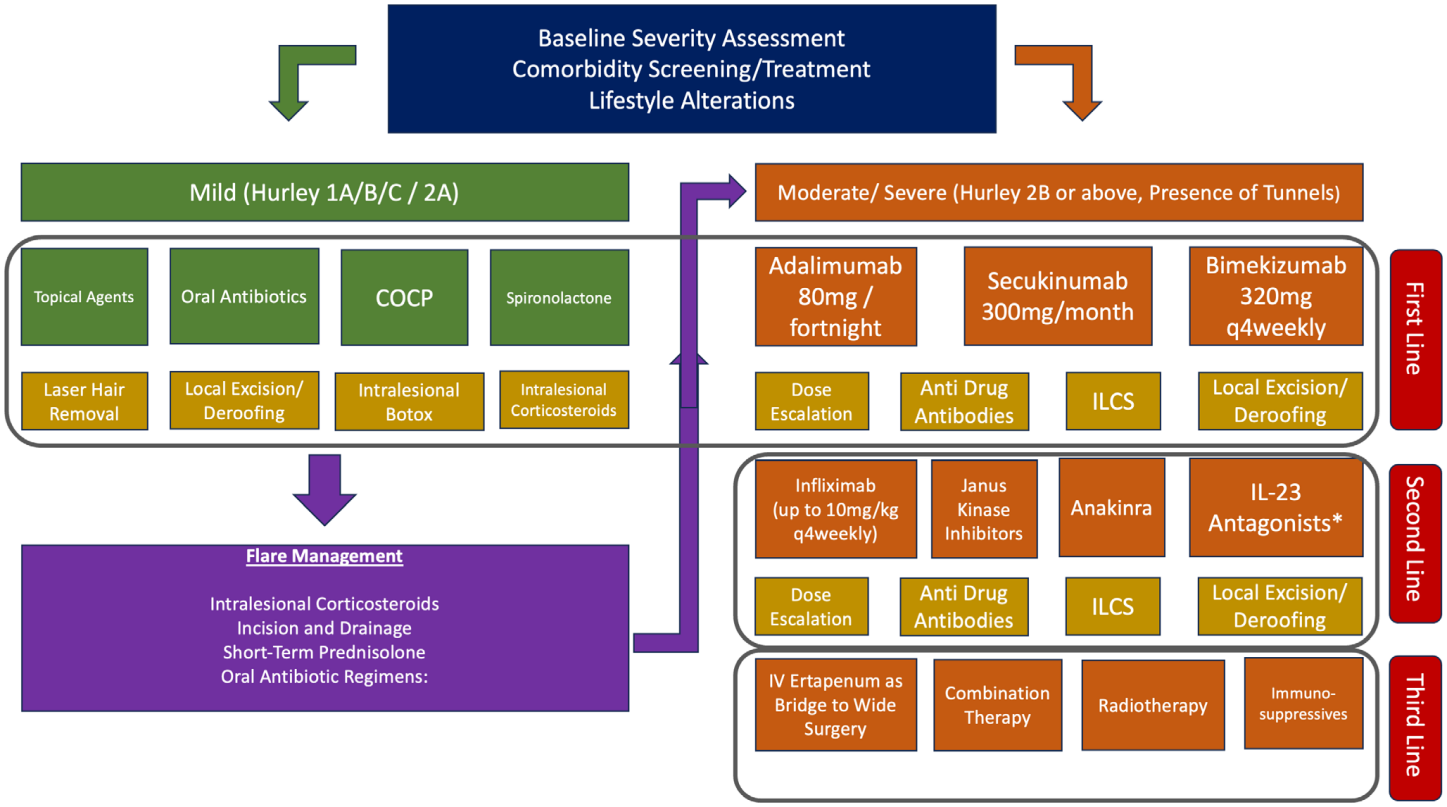
Overall medical management guidelines for Hidradenitis Suppurativa. Baseline disease severity stratification and assessment for appropriate comorbidities should be undertaken with first line therapy determined by the underlying disease activity.

The consensus management approach for mild to moderate HS is presented in Figure 2.

Initial management should include assessment of disease severity, assessment, and evaluation for the presence of comorbidities and a multimodal approach as illustrated in Figure 1. The selection of initial therapeutic agent is dependent upon gender, coexistent medications, patient allergies or intolerances and patient and physician preferences. Topical antiseptics may be used but the level of evidence is low.^67^ (Table 3) RCT evidence suggests in mild to moderate disease relative equivalence of topical antibacterial and oral tetracycline therapy.^68^ Combination therapy with rifampicin clindamycin^69^ (or clindamycin monotherapy^70^) or various antibiotic regimens (including amoxicillin/clauvaulinic acid, co‐trimoxazole, tetracyclines, pristinamycin, levofloxacin) as per the French guidelienes^71^ can be successful in selected cases. For isolated recurrently inflamed lesions—simple excision with primary closure may have a chance of long‐term disease remission.^72^

**TABLE 3:** Summary of Evidence of Therapies in Hidradenitis Suppurativa.

Treatment	Recommendations	Strength of recommendation	Level of evidence
Topical Therapies			
Topical Antiseptics	Mild Disease	C	III
Topical Antibiotics	Mild Disease	C	I
Topical Resorcinol	Adjuvant Therapy	C	IiI
Intralesional Therapies			
Intralesional Steroids	Short Term Flare Management	B	I
Intralesional Botox	Mild Disease/Short Term Flare Management	C	III
Systemic Therapies			
Tetracyclines	Mild Disease/ Short Term Flare Management	A	I
Erythromycin	Mild Disease/ Short Term Flare Management Acceptable In Pregnancy	C	II
Amoxicillin‐clavulanic acid	Short Term Flare Management	C	IV
Linezolid	Short Term Flare Management	C	IV
Rifampicin + Clindamycin	Mild Disease/ Short Term Flare Management	C	I
Rifampicin + Moxifloxacin + Metronidazole	Mild Disease/ Short Term Flare Management	C	III
Clindamycin Monotherapy	Mild Disease/ Short Term Flare Management	C	I
Ertapenum	6 weeks IV Bridging Therapy Prior to Surgery	C	III
Hormonal Contraceptive	Perimenstrual Flaring/PCOS	C	III
Spironolactone	Perimenstrual Flaring/PCOS	C	III
Finasteride	Post‐Menopausal Women	C	III
Metformin	Adjuvant Weight Loss/ IR/DM	C	III
Semaglutide/ GLP‐1R Agonsits	Adjuvant Weight Loss/ IR/DM	C	III
Isotretinoin	Concommitant Acne Congolobata / Comedones/ Dissedting Cellulitis of the Scalp	B	III
Acitretin	Mild–Moderate Disease/ Concommitant Acne	C	III
Biologics (Monoclonal Antibodies)			
Etanercept	Not Recommended	Not Recommended	Not Recommended
Certolizumab	200 mg/400 mg q4w (Option in Pregnancy)	C	III
Golimumab	200 mg q4weekly	C	III
Adalimumab	First Line Agent (40 mg/80 mg weekly)	A	I
Infliximab	Up to 10 mg/kg q4 weekly	B	II
Secukinumab	First Line Agent (300 mg q4weekly/ q2weekly)	A	I
Ixekizumab	80 mg q4weekly‐q8weekly	C	III
Bimekizumab	First Line Agent 320 mg q8weekly/ q4weekly	A	I
Ustekinumab	90 mg q12 weekly	C	III
Risankizumab	360 mg q8weekly (Potentially Useful in Sub‐Population)	B	I
Guselkumab	200 mg q4weekly (Potentially Useful in Sub‐Population)	B	I
Small Molecule Therapies			
Upadacitinib	30 mg daily	A	I

The combined oral contraceptive pill (OCP)^73^ and Spironolactone^74^ are effective in minimising and preventing peri‐menstrual flaring, however spironolactone can be a useful therapeutic agent even in the absence of perimenstrual flaring.^75^ Preventive therapies such as laser hair removal^76^ have anecdotal success, and localised deroofing^77^ can be an alternative to simple excision with good local efficacy. Intralesional botox^78^ and intralesional corticosteroids^79^ have not been validated in placebo‐controlled studies but may be a useful adjuvant in addition to baseline oral therapy. Other useful adjuvant therapies described include 15% topical resorcinol^80^ and cryoinsufflation.^81^

Acute flares^82^ in the mild–moderate population can include antibiotics therapy (such as oral tetracyclines, Amoxicillin and Clavaulinic Acid, or linezolid) along with incision and drainage and ILCS (Intralesional corticosteroids) for acutely painful inflamed lesions.^71^, ^77^, ^79^ Previous studies have attempted to identify oral immunomodulating agents for use in mild–moderate disease sch as Apremilast,^83^ however no significant clinical or molecular alterations have been observed in placebo‐controlled studies.^83^ Whenever mild to moderate disease begins to progress with wider body surface area involvement, or the development of epithelialised tunnels,^84^ progression to therapy for moderate to severe disease should be swiftly embraced (Figure 2).

## MANAGEMENT OF HIDRADENITIS SUPPURATIVA: MODERATE–SEVERE DISEASE

Moderate to severe disease is defined as Hurley stage 2B or above OR the presence of tunnels. Therapeutic options should include systemic biologic therapy, alongside management of inflammatory comorbidities including PCOS, Insulin Resistance etc.

With presence of disease at Hurley Stage 2b/2c or stage 3 (or any disease with the presence of tunnels), therapeutic escalation to moderate to severe disease is essential. It is well documented that the presence of epithelialised tunnels impacts the inflammatory characteristics of disease^89.90^, and is associated with elevated levels of systemic inflammation,^85^ and decreased clinical response to biologic therapies including Adalimumab^31^ and Secukinumab.^86^

Three biologic agents have been shown to be safe and efficacious in moderate‐to‐severe HS, these include Adalimumab,^33^ Secukinumab^34^ and Bimekizumab.^35^ (Table 2). Hence these three agents are listed as first line agents in the treatment of moderate‐to‐severe disease. The selection of which agent is at the clinician's discretion based upon comorbidities (eg the presence of inflammatory bowel disease would be a relative contraindication to IL‐17 inhibition,^87^ and the presence of psoriasis or axial spondyloarthropathy may be an indication for the use of IL‐17 inhibitors^88^).

In the presence of a primary lack of clinical response after 24 weeks of therapy, options include dose escalation (80 mg weekly of Adalimumab,^89^ 300 mg fortnightly for secukinumab^34^ and 320 mg 4‐weekly for Bimekizumab^35^) as well as the additional of adjuvant therapies such as ILCS,^79^ deroofing of persistently draining tunnels^56^ and optimising treatment of existing comorbidities (eg PCOS, insulin resistance etc).

Second line therapies include agents which have completed Phase 2 clinical trials (or large replicated controlled clinical studies) demonstrating efficacy; but are yet to be validated in Phase 3 clinical studies. These include Infliximab,^90^, ^91^ Janus Kinase inhibitors (Upadacitinib),^92^, ^93^ Anakinra^94^ and IL‐23 inhibitors such as Risankizumab^95^ and Guselkumab.^96^ In the case of IL‐23 inhibitors, these agents did not achieve the primary outcome of efficacy compared with placebo response,^95^, ^96^ however translational studies indicate that these agents may be effective in a subset of patients with low BMI and high serum testosterone levels.^97^ Other IL‐23 antagonists such as Tildrakizumab^98^ have been reported in case series but have not been validated in larger placebo controlled trials.^98^ Third line agents include other agents reported in case series or uncontrolled studies including IV Ertapenum^99^ (as a bridge to wide surgical excision or wide regional laser ablative therapy), combination therapy with biologic agents,^100^ traditional immunosuppressive agents such as tacrolimus,^101^ cyclosporine,^102^ methotrexate,^103^ or radiotherapy.^104^ All of these third line agents have been described in cohort studies with low level of evidence.

The use of biosimilar medications^105^ is not opposed in the treatment of HS. Low level evidence exists suggesting a greater secondary loss of efficacy in biosimilar cohorts,^106^ with an increased risk of efficacy loss with switching between biosimilar and originators.^106^ Based upon this evidence, switching between agents is not recommended.

## FLARE MANAGEMENT

Flares are an expected complication of the disease in Hidradenitis Suppurativa. Proactive planning of flare management is essential. Recommended options include incision and drainage, deroofing, ILCS, oral antibiotics or oral prednisolone.

Flares in Hidradenitis Suppurativa frequently often occur even in the presence of effective systemic therapy.^107^ The definition of flare often varies between patients and clinicians.^108^ The strict clinical trial definition of flare is an increase of abscess and nodule count 25% above baseline levels, however for many patients, one new lesion will be considered a subjective flare.^108^ Hence discussion of flare management is essential prior to the onset of flares.

The evidence base for flare management is low given the temporary nature of flares.^109^ Reported approaches include oral antibiotic combinations, oral prednisolone, intralesional steroids, minor surgical procedures such as incision and drainage or de‐roofing.^109^, ^110^ Again, the aim of flare management is to reduce the pain, swelling and distress associated with the acute event.^109^, ^110^ Repeated flares of increasing frequency are often a suggestion of loss of clinical response to the base therapy, and consideration of altering the underlying base therapy should be considered.

## MANAGEMENT OF LOSS OF THERAPEUTIC RESPONSE

48% individuals with HS lose therapeutic response to biologic therapy within 12 months.^111^, ^112^ Management options include anti‐drug antibody testing, dose escalation, adjuvant therapies or switching of agents. Individuals presenting with a secondary loss of clinical response in the setting of a previously effective therapy should be clinically assessed to determine any potentially reversible trigger which may have led to a temporary loss of response^114,116,117^ This can include recent viral infection, rapid weight gain, commencement or cessation of medications or temporary psychological stressors etc.^113^ If no reversible cause is identified, and the loss of response does not spontaneously resolve, consideration of a loss of clinical response should be considered.

In the setting of mild‐to‐moderate disease – loss of response associated with maintaining a Hurley stage 1 or 2A disease consideration would be given for ILCS or localised surgical intervention.^109^ In the setting of Hurley *stage* 2B or above, progression to systemic management should occur. In pre‐existing moderate‐to‐severe patients, loss of response to first line biologic therapy should trigger an assessment for anti‐drug antibodies,^114^ as well as the development of any new comorbidities which may contribute to disease activity (insulin resistance, diabetes etc).^113^ Adjuvant therapies such as ILCS and targeted surgical intervention may rescue the existing therapy, however dose escalation in the absence of antidrug antibodies (and ideally low serum drug levels) may be required. A table of suggested approaches to management of anti‐drug antibodies is presented in Data S1. Unsuccessful management using these methods may necessitate transition to an alternative agent or escalation to second‐line or third‐line therapies.

## MANAGEMENT OF HIDRADENITIS SUPPURATIVA DURING PREGNANCY AND BREASTFEEDING

HS should be optimised as much as possible prior to conception. Decision to continue monoclonal antibody therapies during the 1st and 2nd trimester are based upon a risk–benefit assessment. Certolizumab pegol does not cross the placenta and can be useful during the third trimester. Expected post‐partum flares require aggressive management.

**FIGURE 3:**
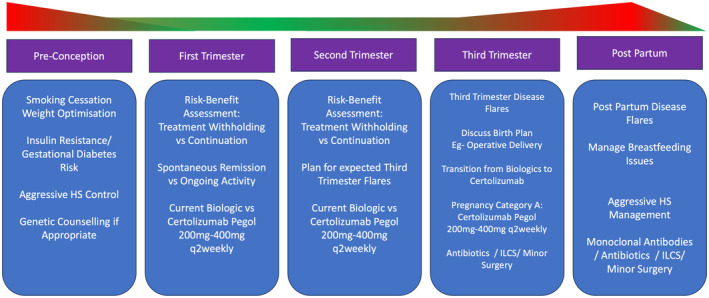
Management of HS during pregnancy and the post partum period: Disease activity commonly flares during pregnancy and consideration to the safety of medications for both mother and foetus are required. Disease optimisation at each stage is required, however, decisions regarding continuation of biologic therapy are based upon a risk–benefit assessment between patient and provider.

HS has a strong hormonal influence^115^ and hence the management of HS during pregnancy and in the postpartum period can be challenging.^115^ The natural history can be variable with some women experiencing spontaneous remission during the first 24 weeks of pregnancy, and others experiencing significant flares.^116^ The third trimester and post‐partum periods tend to be consistent in their predilection for disease flaring due to the large hormonal changes during this time.^116^ The general recommendations for management of HS during pregnancy and post‐partum is summarised in Figure 3. The evidence base for therapies in pregnancy is rather low, and largely based upon cohort studies and expert opinion.^115^, ^116^, ^117^ Pre‐conception optimisation of disease activity is recommended. For mild to moderate disease, patients should be transitioned to medication that is safe during conception and pregnancy. This would include cessation of the contraceptive pill and other medications such as metformin and spironolactone. Antibiotics with pregnancy category A such as erythromycin and cephalosporins can be safely continued if needed. For moderate to severe disease, the use of monoclonal antibodies during the first and second trimesters must be considered on a case by case basis.^118^ The decision to continue monoclonal antibody therapy during pregnancy in HS should be made as a risk–benefit decision between the clinician and the patient. As antibodies are not actively transported across the placenta until the third trimester, in the appropriate setting, biologic therapy can be continued. The premise of continuing therapy during pregnancy is that the excessive inflammatory load is more of a risk to the viability of the pregnancy than the presence of the medication.

The use of oral prednisolone during pregnancy is possible, but again to be determined on a case by case basis. Awareness of the shared pre‐existing risks for gestational diabetes and HS should be acknowledged, and any gestational diabetes managed aggressively. Certolizumab Pegol^119^ is a pegylated TNF‐a inhibitor which does not cross the placenta due to its increased molecular size and absence of an Fc portion. It is considered safe in pregnancy and is an additional option for HS management during the third trimester which does not cross the placenta due to its increased molecular size.^119^

Whilst some cross‐sectional epidemiological evidence suggests that birth outcomes in the setting of HS are suboptimal with increased risk to the foetus and mother,^116^, ^120^ expert opinion is that appropriate medical management of HS can ameliorate these risks.^116^ HS is not a contraindication to vaginal birth or breastfeeding^116^ and mothers should be encouraged to pursue vaginal births or breastfeed according to their preference.^116^, ^121^ Immediately post‐partum, aggressive disease management should be instituted in order to prevent post‐partum flares. Biologic therapy does not enter breastmilk in sufficient quantities after Day 3 post‐partum to be biologically active and the proteins are denatured in the infant gut.^122^

## GUIDELINE LIMITATIONS

These guidelines have intrinsic limitations given their use in the context of the Australian healthcare system. Despite Hurley staging not being recommended, it is still required for Pharmaceutical Benefit Scheme (PBS) application for Adalimumab and Secukinumab despite its limitations in clinical utility. No other biologic therapies are currently reimbursed under the PBS for Hidradenitis Suppurativa. Item codes for surgical procedures in HS do exist, for example wide excision (31245) and sinus excision (30099), however no specific item code for de‐roofing currently exists. Hence financial constraints in accessing therapy do currently limit therapeutic options in most patients with HS in Australia. The hope is these guidelines will stimulate the discussion of further support and access to appropriate therapies for the management of HS.

## CURRENT CONROVERSIES AND FUTURE ENDEAVOURS NEEDED

The current evidence base for therapy in HS is relatively low, with only a small number of agents completing Phase 3 placebo controlled clinical trials.^33^, ^34^, ^35^ Given the relatively low level of efficacy in the context of trials, exploration into disease heterogeneity or the identification of biomarkers which may indicate the likelihood of clinical response would be helpful for practicing clinicians.^23^ Additionally, many newer mechanisms of action (such as B cell antagonists^123^, ^124^) have been used in early‐stage clinical trials. This should provide further insight into disease pathogenesis which may uncover novel therapies and mechanisms of actions specific to HS. Additional evidence pertaining to the ideal timing of surgery in HS, and if long term therapies can prevent progression of disease are also greatly needed.

## FUNDING INFORMATION

Nil.

## CONFLICT OF INTEREST STATEMENT

J.W.F. has conducted advisory work for Janssen, Boehringer‐Ingelheim, Pfizer, Kyowa Kirin, LEO Pharma, Regeneron, Chemocentryx, Abbvie and UCB, participated in trials for Pfizer, UCB, Boehringer‐Ingelheim, Eli Lilly, CSL and received research support from Ortho Dermatologics, Sun Pharma and La Roche Posay. JWF is the Editor‐in‐Chief of the Australasian Journal of Dermatology and a co‐author of this article. They were excluded from editorial decision‐making related to the acceptance and publication of this article. Editorial decision‐making was handled independently by Dr. Kennedy to minimise bias.

## ETHICS STATEMENT

This study has been approved as Negligible or Low risk by the Human Research Ethics Board of the Sydney South West Area Health Service.

## Supporting information


Data S1.

## Data Availability

*The data underlying this article will be shared on reasonable request to the corresponding author*.
