# Supplementary material for: Australasian hidradenitis suppurativa management guidelines
Source: Australas J Dermatol. 2024 Nov 22;66(2):75–89. doi: 10.1111/ajd.14388 (PMC11898165; doi:10.1111/ajd.14388)
Supplement: Supplementary file 1 — Data S1. [file AJD-66-75-s001.docx]

**Australasian Hidradenitis Suppurativa Management Guidelines**

**Supplementary Material**

**Supplementary Figures and Tables:**

Supp File 1: Systematic Review Inclusion and Exclusion Criteria

Supp File 2: PRISMA Flow Diagram

Supp File 3: Delphi Consensus Flow Diagram

Supp File 4: HiSCR responses of monoclonal antibody therapies in HS

Supp File 5: Levels of Evidence for Therapeutic Studies

Supp File 6: GRADE Practice Recommendations

Supp File 7: Disseminated consensus statements

Supp File 8: Items Not Reaching Consensus (Round 1)

Supp File 9: Items Not Reaching Consensus (Round 2)

Supp File 10: Management of Anti-Drug Antibodies

**Supplementary File 1: Systematic Review Inclusion and Exclusion Criteria**

**Supplementary File 2: PRISMA Diagram**

**Identification of studies via databases and registers**

Records removed *before screening*:

Duplicate records removed (n=5582 )

Records marked as ineligible by automation tools (n=2736)

Records identified from:

Medline (n = 3180 )

OVID (n = 4775 )

Web of Science (n = 2990)

Total (n=10945)

**Identification**

Records screened

(n =2627 )

Records manually excluded

(n =1689 )

Reports sought for retrieval

(n =938 )

Reports not retrieved

(n =0 )

**Screening**

Reports excluded (n=107)

Not Regarding Therapy (n=45)

Not Regarding HS (n=62)

Reports assessed for eligibility

(n=938 )

Studies included in review

(n=831)

**Included**

**Search Strategy:**

Databases: Medline, Embase, Web of Science

Dates:1947= Feb 29 2024

Search Terms ("Hidradenitis Suppurativa" OR "Acne Inversa") AND ("therapy" OR "Treatment" OR "Biologics" OR "Surgery" OR "Laser" OR "Intervention")

**Supplementary File 3: Delphi Consensus Diagram**

**Supplementary File 4: HiSCR Responses of various biologic therapies in HS**

| **Medication** | **Target** | **HiSCR-50 Response** | **HiSCR-75 Response** | **Placebo Response** | **Strength of Recommendation** | **Level of Evidence** | **References** |
| --- | --- | --- | --- | --- | --- | --- | --- |
| Adalimumab* | TNF-alpha | 41-58% | 36% | 25% | A | 1A |  |
| Infliximab | TNF-alpha | 60-81% | N/A | N/A | B | 2A |  |
| Secukinumab* | IL-17A | 45% | N/A | 34% | A | 1A |  |
| Bimekizumab* | IL-17A/F | 55-58% | 60% | 28.7% | A | 1A |  |
| Sonelokimab | IL17A/F | 65.7% | 57% | 27.9% | B | 1B |  |
| Upadacitinib | JAK1 | 38.3% | N/A | 28.3% | B | 1B |  |
| Povorcitinib | JAK1 | 88% | 40.7% | 57% | B | 1B |  |
| Lutikizumab | IL1A/B | 59.5% | 45.9% | 35% | B | 1B |  |
| Anakinra | IL-1B | N/A | N/A | N/A | C | 3A |  |
| Remibrutinib | BCR | 72.7% | 42.4% | 34.7% | B | 2A |  |
| Spesolimab | IL-36R | 31.1% | N/A | 17% | B | 1B |  |
| Guselkumab | IL-23 | 55.9% | N/A | 45.2% | B | 1B |  |
| Risankizumab | IL-23 | 46.8% | N/A | 41.5% | B | 1B |  |

**Supplementary Table 5: Levels of Evidence for Therapeutic Studies**

| **Level** | **Type of evidence** |
| --- | --- |
| 1A | Systematic review (with homogeneity) of RCTs |
| 1B | Individual RCT (with narrow confidence intervals) |
| 1C | All or none study |
| 2A | Systematic review (with homogeneity) of cohort studies |
| 2B | Individual Cohort study (including low quality RCT, e.g. <80% follow-up) |
| 2C | “Outcomes” research; Ecological studies |
| 3A | Systematic review (with homogeneity) of case-control studies |
| 3B | Individual Case-control study |
| 4 | Case series (and poor quality cohort and case-control study |
| 5 | Expert opinion without explicit critical appraisal or based on physiology bench research or “first principles” |

**Supplementary Table 6: GRADE Practice Recommendations**

| **Grade** | **Descriptor** | **Qualifying Evidence** | **Implications for Practice** |
| --- | --- | --- | --- |
| A | Strong recommendation | Level I evidence or consistent findings from multiple studies of levels II, III, or IV | Clinicians should follow a strong recommendation unless a clear and compelling rationale for an alternative approach is present |
| B | Recommendation | Levels II, III, or IV evidence and findings are generally consistent | Generally, clinicians should follow a recommendation but should remain alert to new information and sensitive to patient preferences |
| C | Option | Levels II, III, or IV evidence, but findings are inconsistent | Clinicians should be flexible in their decision-making regarding appropriate practice, although they may set bounds on alternatives; patient preference should have a substantial influencing role |
| D | Option | Level V evidence: little or no systematic empirical evidence | Clinicians should consider all options in their decision making and be alert to new published evidence that clarifies the balance of benefit versus harm; patient preference should have a substantial influencing role |

**Supplementary File 7: Consensus Statement Dissemination to Experts**

1. HS Diagnosis should be based upon the modified Dessau Criteria (2 typical lesions in 2 different anatomical areas within a 6month period)
2. Appropriate Disease Assessment in HS include:
   1. Hurley Staging
   2. Refined Hurley Staging
   3. Abscess and Nodule Count (doesn’t count tunnels)
   4. International Hidradenitis Suppurativa Severity Score (IHS4) (counts tunnels)
   5. Sartorius Score
   6. HASI (Hidradenitis Suppurative Area and Severity Index)
3. Appropriate Comorbidity Assessment should include:
   1. Obesity
   2. Smoking
   3. Family History
   4. Inflammatory Arthritis
   5. Inflammatory Bowel disease
   6. Depression/Anxiety
   7. Diabetes/Insulin Resistance
   8. PCOS
   9. Dyslipidaemia
4. Appropriate therapeutic General Measures should include
   1. Smoking Cessation
   2. Weight Loss
   3. Treatment of co-existent Inflammatory diseases
   4. Dietary Changes
   5. Psychosocial Support
   6. Wound Care
5. Appropriate Topical Therapy Should include:
   1. Antiseptic Washes
   2. Topical Antibiotics
   3. Topical Steroids
   4. Resorcinol
6. Intralesional Steroids should be used in the management of HS:
   1. Only for Flares
   2. Consistently long term
7. Oral Antibiotics should be used in the management of HS:
   1. Only for Flares
   2. Consistently long term
8. Hormonal Agents used in the management of HS should include:
   1. OCP
   2. Spironolactone
   3. Finasteride/Dutasteride
9. Metabolic Agents used in the management of HS should include:
   1. Metformin
   2. Semaglutide etc
10. Traditional Immunomodulators/Immunosuppressants used in the management of HS should include:
    1. Oral Steroids
    2. Isotretinoin
    3. Acitretin
    4. Methotrexate
    5. Cyclosporin
    6. Azathioprine
    7. Dapsone
    8. Hydroxychloroquine
11. Biologic Agents used in the management of HS should include:
    1. Etanercept
    2. Adalimumab
    3. Infliximab
    4. Certolizumab Pegol
    5. Secukinumab
    6. Ixekizumab
    7. Guselkumab
    8. Risankizumab
    9. Tildrakizumab
    10. Anakinra
    11. JAK Inhibitors
12. Surgical Intervention used in the management of HS should include:
    1. Deroofing
    2. Wide Excision
    3. Whole Anatomical Area Removal (Plastic Surgeon)
    4. Surgery Alone
    5. Surgery in combination with medical therapy
13. Physical Therapies used in the management of HS should include:
    1. Laser Hair Removal
    2. PDT
    3. Ablative Laser for HS
    4. Cryoinsufflation (tunnels)
14. Free Text Response: Are there any other therpaies which should be included?
15. The overall treatment approach in HS should be:
    1. Steady progression of monotherapy (ie one therapy at a time sequentially) (eg- topicals… then progress to oral antibiotics…. Then progress to spironolactone…. Then progress to Adalimumab…. Then progress to surgery….)
    2. Multimodal concurrent therapy (multiple therapies simultaneously)
16. Screening for Comorbidities in HS: Please complete the following Table.

| **Comorbidity in HS** | **Prevalence in HS** | **Number Needed to Screen** | **Level of evidence** | **Strength of recommendation** | **Should we Screen? (Y/N)** |
| --- | --- | --- | --- | --- | --- |
|  |  |  | | |  |
| Acne vulgaris/conglobata | 4.5% | N/A | II | B |  |
| Dissecting cellulitis of scalp | 9.2% | N/A | II | B |  |
| Pilonidal cyst | 32.6% | N/A | II | B |  |
| Pyoderma gangrenosum | 0.18% | N/A | II | B |  |
| Depression | 26.5% | 108 | II | B |  |
| Anxiety | 18.1% | 2169 | II | B |  |
| Suicidality | 0.014% | 1014 | II | B |  |
| Substance misuse | 4% | N/A | II | B |  |
| Polycystic ovary syndrome | 9% | N/A | II | B |  |
| Obesity | 41.3% | N/A | II | B |  |
| Dyslipidemia | 48.9% | 12 | II | B |  |
| Diabetes mellitus | 10.6% | 49 | II | B |  |
| Metabolic syndrome | 40% | N/A | II | B |  |
| Hypertension | 24.4% | 11 | II | B |  |
| Cardiovascular disease | 2.8% | 18 | II | B |  |
| Inflammatory bowel disease | 1.8% | N/A | II | B |  |
| Spondyloarthritis | 0.9% | N/A | II | B |  |

Concerning the management of HS during Pregnancy and Breastfeeding: please answer Yes/No or provide free text answers.

PRE-CONCEPTION

1. Smoking Cessation and Weight Optimisation are important to minimize HS-related flares in pregnancy
2. Biologic therapies should be ceased as soon as pregnancy is identified-
3. Continuation of monoclonal antibody therapy in the first and second trimester is based on a risk/benefit analysis in the individual patient
4. Given 2/3 of women have a spontaneous improvement in their HS in pregnancy, watchful waiting can be appropriate in some instances
5. Genetic Counselling (except in the presence of a defined autoinflammatory syndrome) is not appropriate for the majority of HS patients

FIRST AND SECOND TRIMESTER

1. Biologic therapy can be continued if the risk/benefit ratio is appropriate
2. Certolizumab Pegol 200-400mg q2weekly should be first line therapy for HS in pregnancy
3. Adjuvant therapies for flares such as antibiotics/ILCS/deroofing can be conducted on a case by case basis
4. Planning should commence for risk of flares in third trimester and post partum
5. Multidiscplinary management with an obstetrician is recommended for severe disease

THIRD TRIMESTER

1. Third trimester flaring should be managed with Cetrolizumab Pegol.
2. Oral corticosteroids/ antibiotics can be used for acute flares after discussion with obstetrician
3. Vulval HS is not a contraindication to vaginal delivery
4. HS does not confer an infection risk to the child

POST PARTUM

1. Aggressive control of HS post partum with biologic therapy / antibitoics etc if required s needed to avoid dramatic flares
2. HS is not a contraindication to breast feeding
3. Biologic therapies are safe in breastfeeding

**Supplementary File 8: Items Not achieving Consensus (Round 1):**

After the first round, consensus was achieved on:

64/67 items for diagnosis and assessment

15/17 items for pregnancy and breastfeeding

13/15 items for comorbidities

The following items which did not meet consensus are listed below:

1. HS Disease assessment should use Abscess and Nodule count (not including tunnels) (57% consensus of NO)
2. Finasteride/Dutasteride should be used as hormonal therapy in HS (52% consensus of NO)
3. Acitretin should be used as therapy for HS (60% Consensus YES)
4. Certolizumab Pegol 200-400mg q2weekly should be first line therapy for HS in pregnancy (50% consensus YES)
5. Third trimester flaring should be managed with Cetrolizumab Pegol. (50% consensus YES)
6. Suicidality should be screened for in HS (60% consensus YES)
7. Substance misuse should be screened for in HS (60% Consensus YES)

**Supplementary File 9: Items Not Reaching Consensus (Round 2):**

| Original Wording | Revised Wording | Consensus at Round 2 |
| --- | --- | --- |
| Acitretin should be used as therapy for HS | Acitretin may be used in specific patients (eg- males, concomitant acne congolobata, extensive comedones etc) | 52% Yes  48% No |
| Certolizumab Pegol 200-400mg q2weekly should be first line therapy for HS in pregnancy | Certolizumab Pegol 200-400mg q2weekly is an option for HS in pregnancy | 52% Yes  24% Yes (if changed)  24% No |
| Third trimester flaring should be managed with Cetrolizumab Pegol | Third trimester flaring may be managed with Cetrolizumab Pegol | 52% Yes  24% Yes (if changed)  24% No |

**Supp File 10: Management of Antidrug Antibodies**

|  | Anti-Drug Antibody Levels LOW | Anti-Drug Antibody Levels HIGH |
| --- | --- | --- |
| Serum Drug Levels LOW | Increase Dose/Frequency | Add Methotrexate/ Increase Dose/Frequency/ Change Medication |
| Serum Drug Levels HIGH | Change Medication | Add Methotrexate/ Change Medication |
